# Childbearing and Delivery in Women With Ulcerative Colitis and Ileostomy or Ileal Pouch-Anal Anastomosis

**DOI:** 10.14309/crj.0000000000000805

**Published:** 2022-06-23

**Authors:** Michele Campigotto, Andrea Braini, Maria Maddalena Casarotto, Saveria Lory Crocè, Renato Sablich

**Affiliations:** 1 Dipartimento Universitario Clinico di Scienze Mediche, Chirurgiche e della Salute, Università degli Studi di Trieste, Trieste, Italy; 2 Struttura Complessa Chirurgia Generale, Azienda Sanitaria Friuli Occidentale, Pordenone, Italy; 3 Struttura Semplice Degenza Ostetrica, Blocco parto e Gravidanza a rischio–Ostetricia e Ginecologia, Azienda Sanitaria Friuli Occidentale, Pordenone, Italy; 4 Clinica Patologie del Fegato, Azienda Sanitaria Universitaria Giuliano Isontina, Trieste, Italy; 5 Struttura Semplice Malattie infiammatorie Croniche Intestinali–Gastroenterologia, Azienda Sanitaria Friuli Occidentale, Pordenone, Italy

## Abstract

No detailed information is currently available about the management of pregnancy and delivery in patients with a stoma after colectomy for ulcerative colitis. We describe the case of a young pregnant woman with terminal ileostomy after toxic megacolon. Episodes of stoma occlusion, determined by the enlargement of the uterus, were treated with endoscopic decompression and daily assumption of oral laxatives, making possible to avoid surgery and carry pregnancy on until caesarean section was performed at week 37. Fertility issues, facing pregnancy with ileostomy rather than with ileal pouch-anal anastomosis, and choice of caesarean section rather than vaginal delivery are discussed.

## INTRODUCTION

The peak incidence of inflammatory bowel diseases (IBDs) occurs during full female reproductive age.^1^ Despite the advances in medical therapy, colectomy is still necessary in about 20% of cases.^2^ The fertility rate in women with ulcerative colitis (UC) seems to be comparable with that of the general population,^3^ but it may decline after pelvic surgery because of adhesions and obstruction of fallopian tubes,^4^ especially after ileal pouch-anal anastomosis (IPAA).^5^ Because a temporary ileostomy is performed in most cases, the question whether pregnancy and childbirth have a better outcome with a stoma or IPAA is a matter of debate.^6^

## CASE REPORT

**Figure 1.**
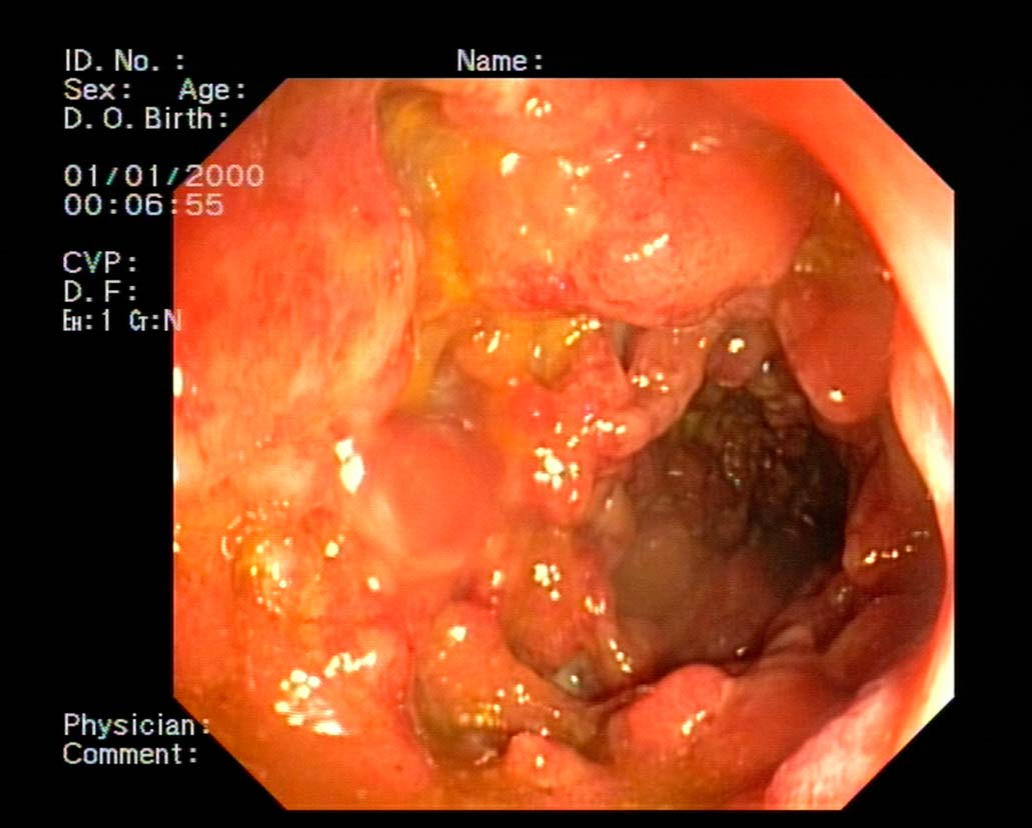
Colonoscopy showing endoscopic Mayo 3 inflammation in the upper colonic segments.

A 27-year-old pregnant woman with a 2-year history of mild ulcerative proctitis was admitted to our unit in August 2016 for a severe flare requiring intravenous steroids followed by rescue therapy with infliximab 5 mg/kg. Concomitant *Clostridium difficile* infection was treated with oral vancomycin. Clinical, biochemical, and endoscopic responses were achieved, with sigmoidoscopy showing an endoscopic Mayo 1 colitis. Despite maintenance with infliximab, symptoms worsened 8 weeks later, leading to miscarriage during the 19th week. Subsequent total colonoscopy revealed mild inflammation up to the proximal sigmoid but severe endoscopic Mayo 3 inflammation in the ascending, transverse, and descending colon (Figure 1). Abdominal X-ray showed features of toxic megacolon. The case was discussed by the multidisciplinary team, and the patient underwent total colectomy with ileostomy in February 2017. She was lost at follow-up but presented to the emergency department in May 2019 complaining of abdominal pain and vomiting and was found to be 16 weeks pregnant. Ultrasound confirmed small intestinal distension upstream, as well as a collapsed and angled segment just a few centimeters from the skin margin, likely caused by postsurgical adhesions and potentially enhanced by the mass effect of the pregnant uterus.

In agreement with colorectal surgeons and obstetrician-gynecologists, endoscopic trans-stomal decompression was performed without sedation using a gastroscope, allowing for drainage of liquid stools and gas. A wire-guided rubber drainage tube was placed 20 cm from the skin margin to overcome the twisted segment. There was no evidence of inflammation or organic lesions. The catheter was connected to a collection bag with immediate emission of about 300 g of semiliquid stools. The drainage was kept in place for 48 hours and used to irrigate the ileum with saline. The dietician prescribed fractionated meals with low fiber intake, abundant oral hydration, vitamins, iron, and protein supplements. Polyethylene glycol 10 mg/daily was also added. After assessing the normal course of pregnancy, the patient was discharged with a scheduled follow-up. The fetus developed regularly, and laboratory workup did not show significant abnormalities. However, the patient needed 7 additional endoscopic decompressions and temporary placement of drainage tube with ever shorter intervals. During week 37 plus 2 days, she underwent an elective cesarean section (CS) and gave birth to a healthy baby.

At the time of writing this report, the patient reports a good quality of life, but because she is planning another pregnancy surgical reconstruction has been delayed. Despite prolonged inactivity, endoanal ultrasound and anorectal manometry revealed preserved anatomy and good sphincter function.

## DISCUSSION

### Pregnancy with ileostomy vs IPAA

The management of pregnant women bearing an ileostomy after total colectomy for UC is poorly described in the most recent guidelines.^7,8^ A small survey mentioned only minor stoma-related complications,^9^ but more important events such as prolapse, urgency, and difficulties in stoma emptying during the third trimester with need for surgical revision have also anecdotally been described.^10^ Only 1 case of ileoscopy to evaluate stoma occlusion during pregnancy is reported in literature.^11^ However, the procedure was only diagnostic, and both endoscopic decompression and guidewire-helped drainage were not described. Our report confirms that, during pregnancy, trans-stomal endoscopy is indicated to detect the cause of stoma occlusion, and it can be safely and repeatedly performed also to decompress the ileum. Surgical placement of a drainage after adhesions removal was reported as an alternative, but it implies surgical risks.^12^

In women with IPAA, pregnancy does not seem to increase the occurrence of pouchitis or pouch strictures. Birth weight, duration of labor, delivery complications, vaginal delivery rates, and unplanned CSs also seem comparable before and after IPAA in the same patients. Nonetheless, long-term complications are reported, including increased daily stool frequency 6-8 months after delivery with and increased rate of fecal incontinence.^5^

In our case, stoma occlusion occurred early during pregnancy, highlighting the problem of potential long-term stoma complications. Endoscopic decompression with temporary placement of a drainage has proved to be simple, effective, and safe. Furthermore, dietary management undoubtedly helped avoided surgery.

### Delivery with ileostomy vs IPAA

Delivery mode in patients with IBD is particularly suitable for a multidisciplinary approach, although it should primarily be dictated by obstetric considerations.^10,13^ It has long been debated whether vaginal delivery (VD) influences continence.^14,15^ In a wide series of healthy patients, fecal incontinence was more common after VD compared with CS (odds ratio 1.65).^16^ This may be attributed to occult sphincter damage after VD as first described using anal ultrasound in the 1990s^17^ and more recently remarked by different authors. Guzmán Rojas et al. reported that 80% of the lesions remain undetected during delivery and are associated with some degree of fecal incontinence in 55% of the cases on a long-term follow-up. No defects were detected in women after CS.^18^ The same considerations may likely be extended to patients with IPAA in whom pouch function may deteriorate in the long run, potentially making the risk of incontinence even higher.

It is also unclear whether to prefer VD vs CS in patients with ileostomy. European Crohn's and Colitis Organization guidelines suggest, with low level of evidence (case series), that presence of a stoma might reduce the threshold for CS, especially if the risk is increased for obstetric reasons. VD can indeed result in anal sphincter damage, preventing effective surgical reconstruction.^7^ However, even in case series where CS is performed in patients with a stoma, no details about technical challenges or short-term/midterm outcomes are described.^19^ In this case, CS was planned after a multidisciplinary evaluation. The occurrence of wound infection was considered negligible, providing that the stoma area had been carefully isolated. Moreover, CS was performed with the presence of a colorectal surgeon in the team to ensure the absence of damage to the rectal stump, which is usually fixed to the anterior abdominal wall. In conclusion, such cases are not extremely rare. In the absence of clear evidence-based guidelines, these conditions must be managed case-by-case with the contribution of a multidisciplinary team. Long-term prospective studies are needed.

## DISCLOSURES

Author contributions: M. Campigotto wrote the manuscript and reviewed the literature. R. Sablich edited the manuscript, provided the image, and revised the manuscript for intellectual content. A. Braini, M. Casarotto, and SL Crocè revised the manuscript for intellectual content. All the authors approved the final manuscript. M. Campigotto is the article guarantor.

Acknowledgments: Special acknowledgments to all the medical and nurse staff of the Gastroenterology and Obstetric Units of “Santa Maria degli Angeli” Hospital, Pordenone, Italy.

Financial disclosure: None to report.

Informed consent was obtained for this case report.
